# The Pathogenesis of Port Wine Stain and Sturge Weber Syndrome: Complex Interactions between Genetic Alterations and Aberrant MAPK and PI3K Activation

**DOI:** 10.3390/ijms20092243

**Published:** 2019-05-07

**Authors:** Vi Nguyen, Marcelo Hochman, Martin C. Mihm, J. Stuart Nelson, Wenbin Tan

**Affiliations:** 1 Department of Cell Biology and Anatomy, University of South Carolina School of Medicine, Columbia, SC 29209, USA; vi.nguyen@uscmed.sc.edu; 2 Department of Otolaryngology, Medical University of South Carolina, Charleston, SC 29425, USA; drhochman@facialsurgerycenter.com; 3 Department of Dermatology, Brigham and Women’s Hospital, Harvard Medical School, Boston, MA 02115, USA; mmihm@partners.org; 4 Department of Surgery, Beckman Laser Institute and Medical Clinic, University of California, Irvine, CA 92612, USA; jsnelson@uci.edu; 5 Department of Biomedical Engineering, University of California, Irvine, CA 92617, USA

**Keywords:** Port wine stain, Sturge Weber syndrome, congenital vascular malformations, MAPK, somatic mutation, laser treatment

## Abstract

Port wine stain (PWS) is a congenital vascular malformation involving human skin. Approximately 15–20% of children a facial PWS involving the ophthalmic (V1) trigeminal dermatome are at risk for Sturge Weber syndrome (SWS), a neurocutaneous disorder with vascular malformations in the cerebral cortex on the same side of the facial PWS lesions. Recently, evidence has surfaced that advanced our understanding of the pathogenesis of PWS/SWS, including discoveries of somatic genetic mutations (*GNAQ*, *PI3K*), MAPK and PI3K aberrant activations, and molecular phenotypes of PWS endothelial cells. In this review, we summarize current knowledge on the etiology and pathology of PWS/SWS based on evidence that the activation of MAPK and/or PI3K contributes to the malformations, as well as potential futuristic treatment approaches targeting these aberrantly dysregulated signaling pathways. Current data support that: (1) PWS is a multifactorial malformation involving the entire physiological structure of human skin; (2) PWS should be pathoanatomically re-defined as “a malformation resulting from differentiation-impaired endothelial cells with a progressive dilatation of immature venule-like vasculatures”; (3) dysregulation of vascular MAPK and/or PI3K signaling during human embryonic development plays a part in the pathogenesis and progression of PWS/SWS; and (4) sporadic low frequency somatic mutations, such as *GNAQ*, *PI3K*, work as team players but not as a lone wolf, contributing to the development of vascular phenotypes. We also address many crucial questions yet to be answered in the future research investigations.

## 1. Introduction

Port wine stain (PWS), also known as congenital capillary vascular malformation, results from differentiation-impaired endothelial cells (ECs) in human skin with a progressive dilatation of immature venule-like vasculatures [1]. PWS initially appears as flat red macules in childhood; lesions tend to darken progressively to purple with soft tissue hypertrophy and, by middle age, often become raised as a result of the development of vascular nodules which are susceptible to spontaneous bleeding or hemorrhage [2,3]. Approximately 15–20% of children with an ophthalmic (V1) dermatomal facial PWS are at risk for Sturge Weber syndrome (SWS) [4], a neurocutaneous disorder with vascular malformations in the cerebral cortex on the same side of the facial PWS [5,6]. Seizures, glaucoma, cerebral cortex atrophy, developmental delay and intellectual impairments commonly occur in infancy and may worsen with age [5,6]. The recent discoveries of somatic mutations in the guanine nucleotide-binding protein, G alpha subunit q (*GNAQ*) (R183Q), phosphatidylinositol 3-kinase (*PI3K*) and activation of mitogen-activated protein kinase (MAPK) and PI3K pathways in skin lesions of PWS/SWS have greatly enhanced our understanding of the pathogenesis of the malformations [7,8,9,10]. In this review, we will summarize our current understanding of the genetic mutations of *GNAQ* and *PI3K* and their roles in activation of MAPK/PI3K in the pathogenesis and progression of PWS/SWS as well as the development of anti-angiogenesis in combination with pulsed dye laser (PDL) for the treatment of PWS/SWS. 

## 2. Clinical Background of PWS/SWS

PWS is one of the most common congenital vascular anomalies, which presents at birth and persists for life [11]. PWS can be mistaken as a bruise because of the initial flat pink appearance of the lesions [12]. The prevalence of PWS is estimated at three to five children per 1000 live births; there are ~1.2 million individuals in the United States and ~26 million people worldwide with PWS birthmarks [13,14,15]. There is no sex predilection, and the inheritance pattern is generally sporadic [16]. PWS does not involute, but rather appears to darken over time due to progressive vascular ectasia [17]. Lesions gradually grow in size and commensurate with the body. Soft tissue or bone hypertrophy, development of vascular nodules as a result of vascular hyperplasia [18,19] occurs in approximately two-thirds of patients by the age of 50 years [20,21]. Soft tissue hypertrophy appears at an average age of nine years (1~29 years), bony hypertrophy begins at an average age of 15 years, while nodules develop at an average age of 22 years (14–53 years) [22]. Approximately 90% of PWS are located on the face, followed by neck, trunk, and extremities at much less frequencies [23,24]. The majority of facial PWS (~90%) are unilateral in a trigeminal dermatomal distribution [25]. 

Depending on the location, size and stage of the PWS, patients may show functional compromise of speaking, eating or vision [22,26]. Furthermore, loss of self-esteem and psychological stress are usually significant clinical problems in the afflicted individuals due to stigmatization and disfigurement [27,28,29]. PWS can also involve the oral cavity mucosa, gingiva, tongue, larynx, nose, neck soft tissue and even the parotid glad, resulting in complications such as macrocheilia, gingival bleeding, dysphonia, parotideal swelling, epistaxis, globus pharyngeus, dysphagia, upper airway obstruction [22,30]. Formation of pyogenic granulomas and the occurrence of eczematous dermatitis can also be observed within PWS [31,32]. The scaling, pruritic, excoriated, and inflammatory conditions associated with eczematous dermatitis can happen solely or most severely within the borders of the PWS [32].

PWS can be diagnosed promptly based on the anatomic location and characteristic appearance of the lesion. However, PWS can exist alone or be associated with many other congenital vascular malformations, such as SWS, Parkes-Weber syndrome, Klippel-Trenaunay syndrome (KTS), Proteus syndrome and arteriovenous malformations (AVM) [33]. Therefore, the co-existence of any other vascular anomalies with PWS, particularly in infants, needs to be examined. Imaging systems, such as Doppler, computed tomography (CT) or magnetic resonance imaging (MRI), can be helpful in determining any possible vascular malformations located in deep tissues, e.g., cerebral vascular malformations or AVM. PWS needs to be differentially diagnosed from infantile hemangiomas (IHs) which usually involute over time [34]. Molecularly, ECs from IHs are Glut-1 positive, but PWS ECs are not [35].

SWS, also called encephalotrigeminal angiomatosis, is a neurocutaneous syndrome with vascular malformations occurring on the face, choroid and leptomeninges [36]. The incidence of SWS is unknown and estimated to be 1 in 20,000-50,000 live births [36]. SWS usually manifests with a facial PWS, but there are scattered SWS cases with presence of PWS in trunk or extremities or no visible PWS present. Approximately 15–20% of children with a facial PWS involving the V1 trigeminal dermatome are at risk for SWS. Moreover, the risk of glaucoma increases up to 50%, which is almost always ipsilateral to the facial PWS [36]. SWS can be diagnosed by typical clinical symptoms, facial appearance of the PWS and brain MRI. However, up to 23% SWS patients may show false-negative MRI results [4]. SWS can cause brain epilepsy, neurological impairment and eye glaucoma [12]. The management options for SWS are limited. The primary goal is to minimize the seizure activity with anticonvulsant medications [36]; when medical management fails, surgical treatment is considered [12,36]. Eye glaucoma is treated to reduce the intraocular fluid pressure [12,36].

## 3. Pathological Phenotypes of PWS/SWS

During the past decade, evidence has systematically documented the pathological characteristics of PWS/SWS, including detailed transmission electron microscopy (TEM) ultrastructure from infantile, hypertrophic and nodular PWS, molecular profiles and upregulation of exocytosis of PWS ECs [1,37,38,39]. These recent data have demonstrated that PWS is a multifactorial malformation, involving not only the vasculature, but also other structures within the dermis. The uncovering of these pathological phenotypes has greatly enhanced our understanding of the etiology and progression of the malformation at the cellular level.

### 3.1. Ultrastructure of PWS Lesions

Strikingly, but not surprisingly, the entire physiological structure of human skin has multiple pathological abnormalities in infantile and early-childhood PWS [38]. Thickening of vessel walls associated with proliferation of pericytes and duplication of basement membranes without significant ectasia are the predominant pathological vascular phenotypes, which occur prior to blood vessel dilation. In addition, degenerated smooth muscle cells and hypertrophied and disorganized collagen and elastic fibers can be observed [38]. This evidence suggests that proliferation of vascular types of cells resulting from developmental impairments is the primary phenotype during pathogenesis of PWS; while blood vessel dilation is the secondary abnormality, which happens during the progression of the malformation.

In hypertrophic and nodular PWS, hyperactive and proliferative ECs, pericytes and fibroblasts are observed [37]. These cells contain large numbers of rough endoplasmic reticulum, stacks of Golgi complexes, vesicles, free ribosomes and distended mitochondria in the cytoplasm [37]. These predominant ultrastructural characteristics represent a highly biosynthetic and metabolic state of these cells [37] and are the typical characteristics of endothelial progenitor cells (EPCs) [40,41]. More recently, we have identified hundreds of differentially expressed (DE) proteins in PWS lesions as compared to normal skin using a proteomics approach. These DE proteins mainly involve in the functions of biosynthesis, membrane trafficking, cytoskeleton and cell adhesion/migration [39], providing the molecular basis for those lesional ultrastructural features [37,38]. Furthermore, TEM studies have shown that there is a significant upregulation of extracellular vesicle exocytosis from PWS blood vessels as compared to control, implying that the extracellular vesicles released by PWS endothelial cells may act as potential intercellular signaling mediators to contribute to the pathogenesis of PWS [39].

### 3.2. Differentiation Impairments of PWS ECs

**Figure 1:**
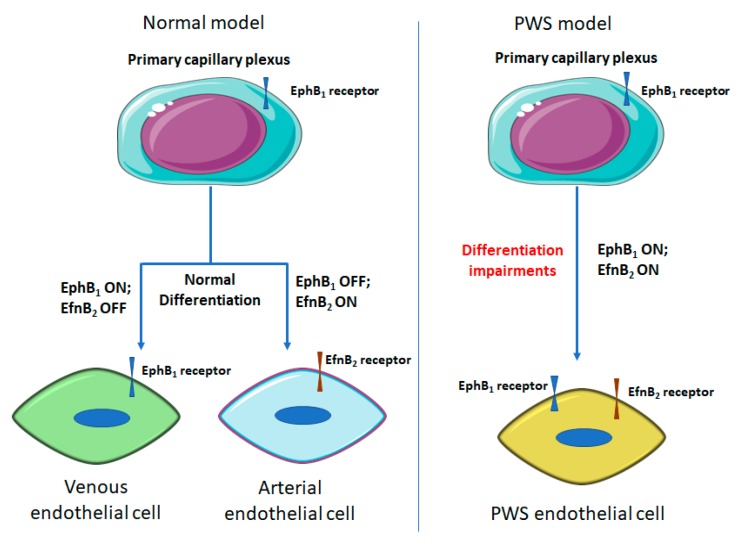
Role of EphB_1_/EfnB_2_ in differentiation of endothelial cell from primary capillary plexus (PCP). EphB_1_ is a biomarker for venous ECs, while EfnB2 is a biomarker for arterial EC. In normal development, mutually exclusive expression of EphB1 or EfnB2 determines dermal arterial or venous differentiation from PCP [43]. In PWS model, both EphB_1_ and EfnB_2_ are co-expressed in ECs, leading to blood vessels with both venous and arterial characteristics.

PWS were usually described as a dilatation of post-capillary venules [42]. However, we have found that there are neither normal arterioles nor venules phenotypically and morphologically in PWS skin after thorough morphological and molecular examinations [1]. PWS blood vessels co-express stem cell markers, CD133 and CD166, as well as venous marker EphB1 and arterial marker EphrinB2 (EfnB2) [1]. During development, both dermal arterioles and venules are differentiated from a primary capillary plexus (PCP) [43,44]. A recent study suggested that turning off EphB1 and switching on EfnB2 is crucial for dermal PCP differentiation into arterioles. In default, the PCP is thought to develop into a vein with consistent expression of EphB1 [43]. It is reasonable to speculate that co-expression of EphB1and EfnB2 in PWS EPCs will inhibit normal differentiation of both arterioles and venules from PCP, resulting in a venule-like vasculature as the predetermined fate of PCP eventually. These PWS ECs are differentiation-impaired EPC-like cells with both venous and arterial molecular properties (Figure 1). Therefore, the current pathoanatomical descriptions of PWS should be re-defined as “progressive dilatation of venule-like vasculatures” [1]. In addition, Efns and Ephs control the development of arterial and venous vasculatures and play a fundamental role in cell-cell interactions [45,46]. Forced co-expression of EphB1 and EfnB2 in normal ECs leads to formation of PWS-like vasculatures in vitro, e.g., large diameter and thick-wall capillaries [1]. These data together suggest that PWS blood vessels are immature capillary vasculatures with aberrant stemness properties and dual venous and arterial identities. PWS is a malformation resulting from differentiation-impaired EPCs in human skin that develop into venule-like vasculatures morphologically and undergo progressive dilatation due to the disruption of EphB1/EfnB2- mediated normal EC-EC interactions. 

### 3.3. Aberrant Activation of MAPK and PI3K in PWS Vasculatures

MAPKs are a family of serine/threonine protein kinases which control a variety of fundamental cellular processes such as proliferation, differentiation, migration, apoptosis, and stress response [47]. MAPKs include the extracellular signal-regulated kinase (ERK), Jun N-terminal kinase (JNK) and stress-activated protein kinases (p38/SAPK) [48]. Both PI3K and MAPK pathways play important roles in the development of the vasculature [49]. The PI3K pathway can induce both angiogenesis and vascular permeability, while MAPK pathway mainly induces angiogenesis [49]. 

There is subsequent activation of various kinases during different stages of PWS [7]: (1) JNKs and ERKs are firstly and consecutively activated in all PWS tissues, which may contribute to both the pathogenesis and progressive development of PWS; (2) AKT and PI3K are subsequently activated and may be involved in hypertrophic PWS blood vessels; and (3) phosphoinositide phospholipase C γ subunit (PLC-γ), PI3K and protein kinase C (PKC) are activated in the most advanced stage of PWS and participate in nodule formation [7,8]. There is a progressive activation of PKCα, PI3K, 3-phosphoinositide dependent protein kinase-1 (PDPK1) and PLC-γ and increased expression of protein phosphatase 2 and diglyceride from normal skin to hypertrophic and nodular PWS [8]. The aberrant activations of these kinases expand from single layer ECs into entire blood vessels stroma and fibroblasts during the progression of PWS nodular formation [8]. 

Activation of MAPK may result from: (1) extracellular stimuli such as cytokines and growth factors. For example, the vascular endothelial growth factor (VEGF)-A and VEGF receptor 2 (VEGFR2) have been found upregulated in PWS [50], which may lead to the activation of VEGFR2 and subsequently activate MAPK; (2) environmental stresses, such as hypertrophied and disorganized collagenous fibers in matrix compositions and changes in blood flow shear forces in dilated PWS vasculatures; (3) alterations in EphB1 and EfnB2 signaling pathways. Both EphB1 forward and EfnB2 reverse signaling can activate MAPK pathways, which leads to a reasonable speculation that the upregulation of MAPK pathways is due to co-expression of EphB1 and EfnB2 in PWS ECs; and (4) genetic somatic mutations, such as *RASA1* and *GNAQ*, which will be discussed below.

## 4. Pathogenesis of PWS/SWS

**Figure 2:**
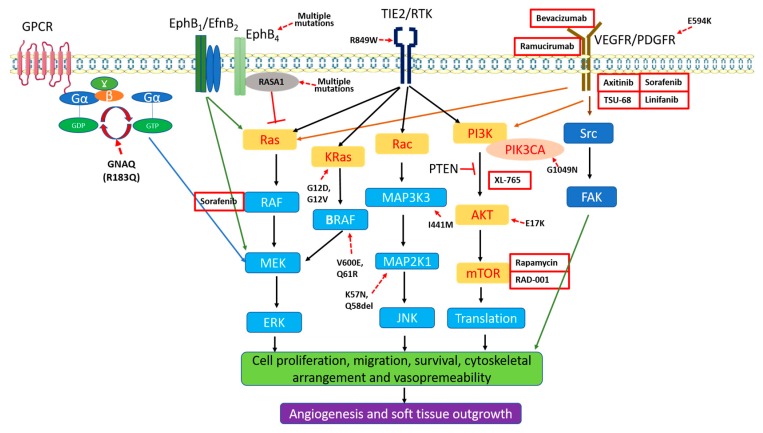
The potential roles of aberrant activations of MAPK and PI3K signaling pathways in pathogenesis of PWS/SWS. Mutations in *GNAQ*, *EphB4*, *RASA1*, *Tie2* and other genes, as well as co-expression of EphB1/EfnB2 lead to an activation of MAPK. Mutation in *PIK3CA* (G1049N) activates AKT/mTOR pathway. Overexpression of VEGF-A and VEGFR2 can activate in MAPK and AKT/mTOR. Altogether, these factors lead to aberrant activations of MAPK and PI3K signaling pathways, thus result in cell proliferation, migration, survival, cytoskeletal arrangement and vasopermeability, eventually causing development of PWS/SWS. Some anti-angiogenesis compounds or antibodies that are under development or have been approved by FDA are listed in the figure for potential treatments of vascular malformations.

Although currently largely elusive, there are at least two major hypotheses, i.e., nerve denervation and genetic mutations, for the pathogenesis of PWS/SWS. The debate on whether the origin of skin lesions of PWS/SWS is purely neural, vascular or neurovascular has continued over decades. PWS usually show a deficiency of nerve innervation that has been speculated as the cause of these abnormal hypervascular skin lesions [51,52,53]. However, confirmatory evidence for this hypothesis has yet to be obtained. Recent discoveries of somatic mutations in the *GNAQ* (R183Q) and *PI3K* in skin lesions of PWS/SWS favor the genetic mutation theory (Table 1) [9,10]. These mutations may lead to the dysregulated vascular MAPK and/or PI3K signaling pathways during human embryonic development thus causing the pathogenesis and progression of PWS/SWS (Table 1) (Figure 2). Herein, we will summarize the current findings regarding the etiology of these malformations.

### 4.1. Nerve Defect

The majority of PWS lesions are unilateral with a typical trigeminal dermatomal distribution. In a study of 310 PWS patients, 32% patients had PWS in the area innervated by the maxillary branch (V2) of the trigeminal nerve, 41% in the combined V1 and V2 branches, 5% in the combined maxillary and mandibular branches (V3), and 10% in all branches [53]. The same study also showed that when PWS involved all three branches there was a significantly higher likelihood of eye and/or central nervous system complications, e.g., SWS [53]. Furthermore, in another study of PWS patients with glaucoma, about 9.3% have V1 trigeminal branch involvement, 30% lesions show V1 and V2 involvements, 5.8% have V2 and V3 branches involvements, and 52% patients show involvements in all three branches [25]. 

Several lines of evidence have shown that PWS vessels lack normal nerve innervations. Smoller et al. reported that S-100 positive nerve fibers were only found in 17% of PWS blood vessels, while the majority (89%) of normal dermal vasculatures or hemangioma tissues had S-100 positive nerve fibers [70]. Rydh et al. showed that nerve defective innervations were only found in pathologically dilated PWS vessels in the middle and deep dermis, but not in other normal skin structures [51]. Selim et al. found that there is a significant decrease in nerve density in all PWS sites as compared to normal skin [54]. Furthermore, PWS ectatic vessels do not respond to epinephrine administration in vitro [71], suggesting defects of sympathetic tonic modulations to PWS blood vessels. Collectively, the absence of nerve innervations to blood vessels may cause a decrease in basal tonus of the vessels and/or a loss of neuronal trophic factors, which contribute to the development of PWS.

However, there are several sets of key evidence yet to be determined to test this hypothesis: (1) which type(s) of peripheral nerves are defective in PWS blood vessels, e.g., sensory, sympathetic or parasympathetic? (2) Which molecules account for this nerve deficiency? and (3) whether nerve deficiency is the primary cause of the vascular phenotypes or a secondary consequence?

### 4.2. Genetic Mutations

#### 4.2.1. *RASA1*

The mutations in *RASA1* were first identified in familial PWS patients with AVM by Eerola et al. [59,60]. *RASA1* mutations have been documented in families with many other vascular malformations, including AVM, SWS, KTS and Parker-Weber syndrome [59,72,73], which suggests that the germline mutations-conferred inherited susceptibility to congenital vascular malformations. *RASA-1* encodes for p120-RasGTP-activating protein (p120-RasGAP), a negative regulator to convert Ras to its GDP-bound form by promoting GTP hydrolysis via its C-terminal catalytic domain [74]. The p120-RasGAP domain participates in protein-protein interactions with Akt, Aurora or RhoGAP, involving signaling regulating the proliferation, migration, and survival of a variety cell types, including vascular endothelial cells [74]. These mutations are usually deletions in the reading frame or mutations in the catalytic domain [59,72,73], which cause a frame shift or premature termination of *RASA1* translation, resulting in a truncated protein with inactivation of the RasGAP domain [59]. The homozygous *RASA-1*^−/−^ mouse is lethal; litters dies at E10.5 from the development defects of vascular system and neuronal apoptosis [75].

#### 4.2.2. *GNAQ* Mutation

The *GNAQ* gene encodes the alpha subunit of heterotrimeric G protein (Gαq) belonging to the membrane bound guanosine triphosphatase (GTPase) family [57,76]. Heterotrimeric G protein associates with G coupled protein receptor (GCPR). Upon a ligand binding to GCPR, it activates the receptor, causing a confirmation change in the G protein, and exchanges in GDP to GTP in Gαq, thus leading to the dissociation of the α subunit from the β and γ subunits which activates downstream signaling pathways, including MAPK [76]. The hydrolysis of GTP is an important on/off switch of the G protein. In other words, Gαq is activated when it bounds to GTP, and it is inactivated when it bounds to GDP [76]. 

Recently, Shirley et al., first found that the somatic mosaic mutation in the *GNAQ* gene (c.548G→A, p.Arg183Gln) (R183Q) in PWS blood vessels in SWS with a low mutation frequency [9]. This mutation was confirmed from skin and brain PWS/SWS ECs by several other studies [37,54,55,57]. We and Cuoto et al. further showed that the *GNAQ* (R183Q) is primarily present in blood vessels using different approaches, e.g., laser capture microscopy or flow cytometry to isolate PWS ECs for mutation analysis [56,58]. We also showed that this mutation is present in connective tissues, hair follicles and glands in PWS, suggesting that pluripotent cells with *GNAQ* (R183Q) may give rise to multi-lineages in PWS [58]. However, the average mutation frequency of *GNAQ* (R183Q) in PWS skin blood vessels is ~7–8% (Table 1) [56,58], which is not significantly augmented as compared to those obtained from the whole lesional tissues (Table 1) [9]. This result raises the question of the role of *GNAQ* (R183Q) in the development of vascular phenotypes of PWS. The average mutation frequency of *GNAQ* (R183Q) in SWS brain ECs is enriched to 18~27% (Table 1) [56,57], suggesting its potential primary role in the pathogenesis of cerebral vascular malformations in SWS. 

GNAQ participates in the MAPK pathway [76,77]. Another *GNAQ* somatic mutation (Q209L) is found in uveal melanoma and blue naevi [77]. This mutation (Q209L) resides in the Gαq kinase catalytic domain and causes an over-activation of ERKs and AKT. Biochemical data shows that GNAQ (R183Q) mutation is located GTP binding domain and induces very minimal ERK activation [77,78,79]. In a silico protein-interaction model, it is proposed that the R183G mutation lost two hydrogen bonds between the Gαq and GDP molecules as compared to native Gαq model [80]. In that case, the R183Q mutation causes instability in inactivation of Gαq [80]. Therefore, the GNAQ (R183Q) likely plays a supportive role in maintaining the consecutive activation status of Gαq which has been activated by other stimuli or factors; while GNAQ (R183Q) alone is unlikely able to activate Gαq. Nevertheless, the close link between GNAQ (R183Q) and strong MAPK activation in PWS has yet to be determined. It is likely that GNAQ (R183Q) and alterations in other genes, such as PI3K, RASA1, together contribute to the progression of PWS. 

#### 4.2.3. PI3K and Other Mutations

A somatic mutation (G1049N) in the phosphatidylinositol-4, 5-bisphosphate 3-kinase catalytic subunit alpha (*PIK3CA*) gene has been found in PWS nodular lesions [10]. In an in vitro experiment, human umbilical vein endothelial cells (HUVEC) expressed with the PIK3CA (G1049N) had faster proliferation rates than normal HUVEC cells [81]. Interestingly, in an EC spheroid assay, HUVEC cells expressing PIK3CA (G1049N) formed capillary like structures without VEGF-A [81]. Therefore, the *PIK3CA* (G1049N) gene can cause hyperproliferation of ECs, which may lead to PWS hypertrophy and nodule formation. Somatic PIK3CA mutations have been found to cause congenital lipomatous overgrowth with vascular, epidermal, and skeletal anomalies (CLOVES) syndrome [65]. Besides the somatic mutation in the *PIK3CA* gene, many other novel somatic mutations have been found in PWS such as *SMARCA4*, *EPHA3*, *MYB*, and *PDGFR-β* [10]. Several somatic mutations in MAPK related genes, such as MAP kinase kinase 1 (*MAP2K1* or *MEK1*), MAP kinase kinase kinase 3 (*MAP3K3*, or *MEKK3*), *EPHB4* and *TEK*, have been found in congenital vascular malformations [63,64,66,82]. However, the detailed roles of these genes in vascular formations have yet to be studied. 

Recently, Al-Olabi et al. identified many mutations in genes related to MAPK pathways, such as *KRAS*, *NRAS*, *BRAF* and *MAPK2K1*, in nine of 25 patients with fast flowing and 10 of 135 patients with slow flowing vascular malformations, including PWS [62]. Particularly, those mutations in *MAP2K1* in exon 2 are predicted to destabilize the inactive form of the kinase [62]. The specific mutations of MAP2K1(K57N), MAP2K1(Q58del) and BRAF(V600E) show strong activation on ERK, which may account for significant activation of the MAPK pathway in those sporadic vascular malformations [62]. Furthermore, the authors showed that BRAF inhibitor vemurafinib can restore the blood flow in AVM-BRAF mutant zebrafish model [62]. 

## 5. Anti-Angiogenesis Therapies for Skin Lesions of SWS/PWS

The current treatment of choice for PWS is pulsed dye laser (PDL), a treatment based on the concept of selective photothermolysis to destroy subsurface-targets without injuring adjacent normal tissue by thermal damage [83]. The PDL wavelength (595 nm) is preferentially absorbed by hemoglobin in blood vessels and converted into heat, thus leading to necrosis of the blood vessel walls. The dynamic skin cooling technology can prevent injury from light absorption by melanin by selective cooling of the epidermis, while the temperature of the PWS blood vessels remains unchanged during PDL [84]. Furthermore, dynamic skin cooling can help reduce the complications such as dyspigmentation or scarring when higher light dosages of PDL are used to expedite PWS lesion clearance [85].

The PDL produces reasonable blanching in patients by effectively destroying superficial PWS blood vessels (≈<300 µm below skin surface) [84,85,86,87,88,89,90]. However, PDLs cannot achieve the critical core temperature necessary to irreversibly destroy blood vessels seated at deeper locations (>300 µm). In order to target blood vessels situated deep in the skin, other vascular laser wavelengths have applied broadly in clinical treatments, including alexandrite 755 nm, diode 800–940 nm and Nd: YAG 1064 nm [16,21]. In addition, photodynamic therapy (PDT) using a photosensitizer in combination with light has been also used in infantile and adult PWS lesions [91]. 

Currently, the vast majority of PWS patients receive a large number (>20) of treatments with marginal success [92,93,94]. However, complete clearance occurs in <10% of patients treated [92,93,94]. Many factors contribute to incomplete PWS blanching such as diameter, depth, wall thickness of blood vessels, skin pigmentation type, hypertrophied or nodular lesions [21]. Regeneration of PDL-photocoagulated blood vessels can cause PWS redarkening or treatment failure [95,96]. Epidermal melanin limits the light dosage that can be safely applied and reduces light delivery to targeted PWS vessels. When the PWS blood vessels are too small or too large, heat cannot be confined to or fill the entire lumen [97,98,99]. Heterogeneity of PWS blood vessel diameter and depth also limits the effectiveness of laser treatment. Different anatomical locations of lesions respond to PDL differently [24]. For example, centrofacial lesions and lesions involving the V2 dermatome respond less favorably than lesions located elsewhere on the head and neck [24].

The regeneration and revascularization of PWS blood vessels induced by laser treatment, often occurs within one month after laser exposure, and is one of the crucial causes of inadequate clinical outcome. As stated by Phung and Nelson et al. [96], “the laser does what it is supposed to do, namely, cause blood vessel wall necrosis. Regrettably, the body also does what it is supposed to do, namely, repair the laser-induced damage.” Therefore, PDL in combination with anti-angiogenic drugs will lead to a better efficacy than PDL alone. PDL-induced local hypoxia leads to upregulation of hypoxia-inducible factor 1-alpha and VEGF [100,101,102], causing activation of angiogenesis pathways including phosphorylation of the mammalian target of rapamycin (mTOR) and p70S6 kinase. As a result, more angiogenic genes are transcribed and translated, leading to reformation and reperfusion of blood vessels. We attempted to block this pathway using angiogenic inhibitors, such as rapamycin (RPM) and axitinib [103,104,105,106], post-PDL treatment. In rodent skin, both topical RPM and axitinib have shown effective inhibition of the early stage of angiogenesis induced by PDL, but blockage of the late stage of angiogenesis proved to be ineffective [105,106]. 

Clinical studies of topical anti-angiogenic drugs have been performed. Topical timolol with PDL failed to significantly improve the efficacy of PDL treatment of PWS [107]. Imiquimod and PDL showed enhanced blanching of PWS compared to controls [108]. Oral or topical RPM exhibited an improvement of PWS lesion blanching in some PWS patients [109,110,111,112]. 

## 6. Conclusions and Future Directions

The evidence accumulated from the past decade has greatly enhanced our understanding of the pathogenesis and progression of PWS/SWS. This knowledge can be categorized into four aspects: (1) the fundamental pathological and histological phenotypes of a variety of cell types in PWS/SWS lesions; (2) the molecular profiles of PWS vasculatures; (3) the germline and somatic mutations in PWS/SWS lesions; and (4) development of new treatment options, such as the expansion of light-based treatments from PDL to PDT. These data have provided cellular, molecular and genetic support for several new concepts of PWS/SWS: (1) PWS/SWS is a multifactorial malformation involving not only the peripheral vascular system, but the entire physiological structure of human skin; (2) PWS ECs are differentiation-impaired ECs showing with a progressive dilatation of immature venule-like vasculatures; (3) the primary contributing signaling pathway to the pathogenesis and progression of PWS/SWS is the dysregulated vascular MAPK and/or PI3K signaling pathways which occurs during human embryonic development (Figure 2); (4) somatic mutations, such as *GNAQ*, *PI3K*, are team players but not as a lone wolf. They may coordinate together and/or with predisposing germline mutations, such as *RASA1*, leading to a vascular phenotype; and (5) laser-based treatments in combination with anti-angiogenic drugs, including anti PI3K or MAPK, have shown several promising pre-clinical and clinical results.

However, there are several crucial gaps that urgently need to be filled in future studies. (1) The decades-long question, e.g., whether the origin of skin PWS/SWS is purely neural, vascular or neurovascular, has yet to be solved. The crucial factors underlying the impaired neurovascular interactions need to be determined. The unilateral distribution of lesions and nerve defects in lesional vasculatures demonstrate that PWS/SWS are types of cutaneous neurovascular malformations. Whether those somatic mutations, such as GNAQ (R183Q), can cause both the neurological and vascular phenotypes are largely unknown, thus urgently need confirmatory evidence. Furthermore, the basic knowledge gaps, such as which types of peripheral nerves are defective, remain unknown. These long-standing questions need a thorough characterization in the future. (2) Current data support the theory that somatic mutations such as *GNAQ* are the primary cause of the pathogenesis of PWS/SWS. However, there are many key questions yet to be answered. There is no direct link between these somatic mutations and the clinical phenotypes of PWS/SWS or other congenital vascular malformations. There is no existing animal model carrying any of these mutations that can replicate a portion of the clinical phenotypes seen in congenital vascular malformation patients. Except *GNAQ*, mutations in other genes such as *KRAS*, *NRAS*, *BRAF*, *PIK3CA*, *EPHB4* and *MAPK2K1* are only found in a very small subpopulation of patients and many are with relative low mutation frequencies, which raise the following questions: (a) whether these sporadic mutations are only the consequences or just by-products of progression of the malformations, but not the primary causes? (b) What will be the real biologically functional outputs of such low level frequencies of these sporadic mutations, if there are any in vivo? They are unlikely working as a lone wolf, but rather more like team players. They may also behave as latent software “bugs” which can be triggered to be functional upon certain extrinsic and/or intrinsic stimuli. There is a notion that germline mutations such as *RASA1* plus a “second hit” of postzygotic mutations, such as *EphB4*, *GNAQ*, *PIK3CA*, *MAPK2K1*, *Tie2*, etc., leads to the development of a vascular phenotype. However, the majority of PWS/SWS are non-inherited. Therefore, this concept may only explain a very limited subpopulation of patients. The *GNAQ* mutation is so far the only somatic mutation that has been reported consistently in the majority of PWS/SWS patients (~60 to 90% in multiple reports, Table 1). However, many biochemical evidence gaps need to be understood to link this mutation and their potential functions in transforming ECs. For example, there is no data showing how the GNAQ (R183Q) can change the function of normal ECs such as proliferation, migration, and survival in vitro. A transgenic mouse model with *GNAQ* (R183Q) knock-in in ECs may be necessary to reveal the function of this somatic mutation in the development of PWS/SWS. (3) The molecular mechanism underlying co-expression of EphB1 and EfnB2 in PWS ECs during development remains unclear. Whether the dysregulation of EphB1/EfnB2 is the primary cause to the vascular phenotypes of PWS or the consequence of those genetic mutations is unknown. What the main signaling outputs of co-expression of EphB1/EfnB2 in PWS ECs and their roles are in vascular phenotype development also need to be addressed. Furthermore, whether the dysregulation of EphB1 and EfnB2 is only present in PWS or also in other types of congenital vascular malformations such as IHs is yet to be answered. (4) There is still lack of data to affirm that the aberrant activation of MAPK and PI3K is the primary cause to the PWS/SWS, although many lines of recent evidence have provided strong leads to this direction. An animal model with phenotypes of vascular malformations will be essential to address this question. Furthermore, the possibilities of involvement of non-MAPK/PI3K signaling in the pathogenesis of PWS cannot be excluded as well. Finally, (5) the inadequate outcome of laser treatment for skin lesions of PWS/SWS is a clinical barrier that requires a solution. Laser treatment in combination with anti-angiogenic drugs provide a promising strategy, but more effective anti-MAPK or PI3K compounds need to be evaluated. There are many anti-angiogenesis compounds or antibodies that are under development or have been approved by FDA which can be potentially used for treatments of vascular malformations (Figure 2). Another concept is to design small molecules or compounds that can specifically target those mutations such as GANQ, BRAF, PIK3CA or MAP2K1. For example, a drug could recognize the specific sequence mutation and bind to GNAQ, then release a signal which inhibits the MAPK pathway, which would be a radical change in the therapeutic paradigm for a personalized medicine. 

## Figures and Tables

**Table 1:** Mutations involving PWS/SWS or associated vascular malformations.

Gene	Mutations	Mutation Frequency Ranges (%)	Average Mutation Frequency *(%)	Positive Rate in Patients	Diagnosis or Sample Resource	Refs
*GNAQ*	R183Q	1.1–18	3.98 ± 3.84	23 of 26	PWS/SWS	[9]
		1.73–7.42	3.86 ± 1.91	9 of 12	PWS	[54]
		3.6–8.9	5.59 ± 1.82	12 of 15	SWS	[55]
		1.9–11.1	5.56 ± 2.65	8 of 13	PWS/SWS	[56]
		2.8–11.3	7.05 ± 6.01	2 ^#^	PWS, skin EC ^&^	
		7.6–42.9	27.35 ± 17.75	4 ^#^	SWS, brain EC ^&^	
		14.7–21.0	17.85 ± 4.45	2 of 2	SWS, brain EC ^&^	[57]
		3.16–12.38	7.85 ± 4.18	6 of 10	PWS, skin BV ^&^	[58]
		2.67–22.17	8.81 ± 7.64	4 of 10	PWS, HG/CT ^&^	
*RASA1*	*RASA1*c.475_476delCT, *RASA1*c.512delT, *RASA1*c.1579_1582delGTCT, *RASA1*c.2336_2337delGC, Q446X, and C540Y	n.a.	n.a.	6 of 17	Familial PWS-AVM	[59,60]
	58 distinct mutations			68 out of 100		[61]
*KRAS*	G12D	2–30 (skin)	16.25 ± 15.33	4 of 160	High/Low flow VM	[62]
	G12V	3–5 (skin)	3.67 ± 1.15	3 of 160		
	Q61H	5	5	1 of 160		
*MAP2K1*	K57N	2–7	4.50 ± 3.53	2 of 160		
	Q58_E62del		4	1 of 160		
	F53_Q58del		6	1 of 160		
*BRAF*	V600E		26	1 of 160		
	Q61R		7	1 of 160		
*MAP3K3*	I441M	5.5–19.3	11.13 ± 5.52	6 of 10	VVM	[63]
*TEK*	L914F	4.66–48.32	20.34 ± 14.61	24 of 57	hereditary mucocutaneous VM	[64]
	Y897H, Y897S, Y897F, Y897C, R915C, R915L, S917I	4.55–34.90		4 of 57		
*PIK3CA*	E542K	6–8	7.00 ± 1.41	2 ^#^	GLOVES/PWS/AVM/LM/VM	[65]
	C420R	3–11	7.00 ± 5.65	2 ^#^		
	G1049N		5	1 ^#^	PWS	[10]
*SMARCA4*	E514Q		7			
*EPHA3*	S456C		7			
*MYB*	G349R		10			
*PDGFR-β*	E594K		6			
*EPHB4*	47 distinct mutations		n.a.	54 of 365	PWS-AVM	[66]
*Tie2*	R849W		n.a.	2 families	inherited VM	[67]
*AKT1*	E17K	3.6–51	22.43 ± 16.77	26 of 29	Proteus syndrome	[68]
*GNA11*	R183C	5.3–9.6	7.45 ± 3.04	2 of 8	Vascular skin lesion of PPV	[69]
	R183Q	5.0–6.4	5.70 ± 0.99	2 of 8		

*, mean ± s.d.; the data was obtained and analyzed from the original reports. ^#^, number of positive patients; n.a., not available. ^&^, Mutations were determined in brain or skin ECs, blood vessel (BV), hair follicle/gland (HG), or connective tissue (CT). Abbreviations: LM, lymphatic malformation; VM, venous malformation; AVM, arteriovenous malformation; VVM, verrucous venous malformation; PPV, phakomatosis pigmentovascularis.
